# Multiple soil map comparison highlights challenges for predicting topsoil organic carbon concentration at national scale

**DOI:** 10.1038/s41598-022-05476-5

**Published:** 2022-01-26

**Authors:** C. J. Feeney, B. J. Cosby, D. A. Robinson, A. Thomas, B. A. Emmett, P. Henrys

**Affiliations:** 1 UK Centre for Ecology and Hydrology, Environment Centre Wales, Bangor, UK; 2 UK Centre for Ecology and Hydrology, Library Ave, Bailrigg, Lancaster, UK

**Keywords:** Biogeochemistry, Carbon cycle

## Abstract

Soil organic carbon (SOC) concentration is the fundamental indicator of soil health, underpinning food production and climate change mitigation. SOC storage is highly sensitive to several dynamic environmental drivers, with approximately one third of soils degraded and losing carbon worldwide. Digital soil mapping illuminates where hotspots of SOC storage occur and where losses to the atmosphere are most likely. Yet, attempts to map SOC often disagree. Here we compare national scale SOC concentration map products to reveal agreement of data in mineral soils, with progressively poorer agreement in organo-mineral and organic soils. Divergences in map predictions from each other and survey data widen in the high SOC content land types we stratified. Given the disparities are highest in carbon rich soils, efforts are required to reduce these uncertainties to increase confidence in mapping SOC storage and predicting where change may be important at national to global scales. Our map comparison results could be used to identify SOC risk where concentrations are high and should be conserved, and where uncertainty is high and further monitoring should be targeted. Reducing inter-map uncertainty will rely on addressing statistical limitations and including covariates that capture convergence of physical factors that produce high SOC contents.

## Introduction

Globally, soils represent the largest reservoir of organic carbon in the terrestrial biosphere, storing at least three times as much carbon as is found in vegetation or the atmosphere^1,2^. Soil organic carbon (SOC) is the main constituent of soil organic matter (SOM)^3^, and underpins a range of soil properties as well as acting as both a nutrient store and energy source for organisms^4–6^. SOC is widely recognised as *the* indicator of soil health^7^. Managing SOC well is therefore critical, preventing carbon loss to the atmosphere, while keeping it in the soil to maintain or enhance soil properties to support food production, conserve biodiversity and maintain the land’s capacity to buffer changes from environmental stressors such as floods and droughts^6^. Increasing SOC is seen as one way of enhancing soil properties to better deliver ecosystem services such as crop production, particularly within relatively carbon-poor mineral soils^1,6,8,9^. Cutting net losses of carbon dioxide to the atmosphere^10,11^ is important, especially from peats where SOC is most abundant, and as organic carbon losses are known to occur more rapidly from soils with an already high standing carbon stock^12^. This is made all the more important by the fact that approximately one third of all soils are degraded which has triggered a significant loss of SOC stocks worldwide^13^. Enhancing our understanding of biophysical drivers of SOC storage to reverse global-scale degradation trends relies on soil monitoring efforts at national scales and larger and was a priority for action identified by the United Nations Intergovernmental Technical Panel on Soils in the ‘Status of the World’s Soil Resources’ report^13^.

Field surveys, such as the UKCEH Countryside Survey^14^, the EU-wide LUCAS soil survey^15^, and global databases like WoSiS^16,17^, offer rich repositories of soil properties. However, many regions of the world cannot be surveyed due to the costs involved, and even in surveyed regions, vast areas of unsampled soil can separate survey points^18^. Remote sensing may offer some utility for monitoring SOC dynamics, though this relies on estimating SOC contents from relationships with covariate proxies such as vegetation and elevation, and without sophisticated process understanding, may merely point to where SOC is likely to decline or accumulate^19^.

Digital Soil Mapping (DSM) offers a powerful tool for modelling the spatial distribution of SOC content at national scales and larger. Generally, DSM proceeds from collecting a database of representative soil carbon observations over the area concerned along with relevant covariates. Information on a subset from the observational database is used to quantify empirical relationships between covariates and any dependence structure that exists in observations to calibrate a spatial prediction function. From this, interpolation and/or extrapolation of the prediction function across the entire area of interest is performed, followed by validation using existing or independent observations^20^. The prediction function in question could be based on a pedo-transfer rule, a statistical function generated via machine learning for instance, or may involve the use of geostatistical methods such as ordinary kriging^21–23^. In more recent years with the proliferation of DSM, several studies have created ensemble maps (based on averaging multiple harmonised primary maps) that have been shown to be more accurate than using an individual published map alone^24–26^.

Great Britain (GB) is an ideal national scale observatory for testing SOC detection and mapping. It lies on the latitudes where SOC increases from low levels in the south (~ 1%), to high levels in the north (100%)^27^. Moreover, it has methodologically coherent state and change SOC data spanning 40 years^28^. SOC is dynamic and sensitive to multiple biophysical drivers across spatial and temporal scales^8^, including climate variables such as temperature and moisture^29–31^, land cover and land-use^32–35^, elevation and other topographic variables^36,37^, latitude^27^, and soil mineralogy^38,39^. These, and a varied parent material, are also represented across GB. The proliferation of large-scale soil surveys and databases, remotely-sensed covariates, and a wide range of DSM techniques have led to increased availability of published maps of SOC concentration in the topsoil (up to 30 cm) in GB. While each of these maps may have been validated individually against observations, no attempt to date has been made to compare published SOC concentration maps with each other. This is an important limitation as predictions from each of these maps may differ substantially from one to another, creating uncertainty as to where degradation is likely prevalent or where there may be the greatest potential to sequester additional SOC. This will affect the delimiting of boundaries between mineral and organic soils where strategies will differ. On the other hand, each of these maps may exhibit some common form of error or poor model performance which may hinder efforts to develop ensemble maps that are more accurate than an individual primary map. Further, calibration and evaluation of models of carbon exchanges between the land surface and atmosphere depends on accurate inventories of SOC concentration^40^. Thus, a comparison of existing SOC concentration map products is an important preliminary step towards demystifying uncertainties in SOC concentration prediction for future model development and land management planning.

In this study, we obtained eight maps of topsoil SOC concentration covering GB and compared their SOC concentration predictions, identifying areas of (dis)agreement and their scale. Next, we compared each map with the UKCEH Countryside Survey 2007 (CS 2007) observations, before stratifying GB into smaller geographical units that represent national-scale controls on SOC concentrations and comparing map predictions with observations at these smaller spatial scales. Here, we demonstrated how each map compares with one another in predicting SOC concentrations in different environments, illustrating the strengths and limitations of the models used to generate SOC predictions as described in their underlying literature.

## Results

### Inter-comparison of eight topsoil organic carbon concentration maps

**Figure 1:**
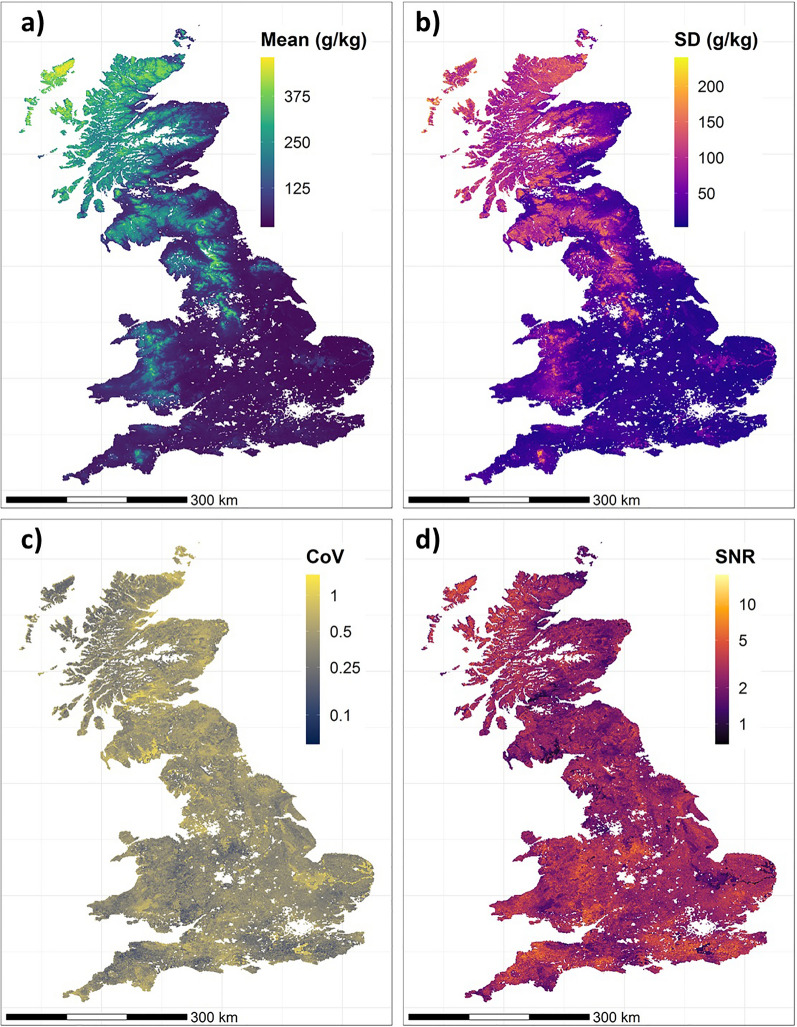
Maps of the (**a**) mean topsoil SOC concentrations of all eight maps; (**b**) standard deviations of the mean (SD); (**c**) coefficient of variation values (standard deviation/mean); and (**d**) the signal to noise ratios (the reciprocal of the coefficient of variation i.e. mean/standard deviation). Note here that statistics were calculated after each of the 8 maps were modified to harmonise them to a common spatial extent, resolution and units of SOC concentration. Values are calculated only for those 1 km grid cells that contain data from all the topsoil SOC concentration maps. White areas indicate where there are no data for at least one of the maps, including urban areas and littoral broad habitats with little topsoil and parts of Scotland (excluded from CSGB-AIC due to the low spatial representation of CS 2007 points in montane broad habitats).

Taking the mean and standard deviation of SOC concentration predictions at each grid cell where data are available for all maps (Table 1 [see “Methods”]; Table S1) allows us to see where there is general agreement or disagreement between map estimates. There appear to be common trends in the spatial distributions of mean and standard deviation, with values increasing along a general southeast to northwest gradient (Fig. 1a and b). As standard deviations largely increase commensurately with the means, the coefficient of variation values tend to be relatively low across much of the country (typically between 0.25 and 0.5) (Fig. 1c). However, there are pockets of topsoil, such as in the East Anglian Fens and parts of southern Scotland where there is considerable disagreement between some of the maps (coefficient of variation ≥ 1). Signal to noise ratio values are highest in the southern half of GB and the England-Wales border region, indicating a high level of conformity among the eight maps in these areas (Fig. 1d). However, values are lowest in places such as the East Anglian Fens, parts of southwest England and the low-lying areas of Scotland, showing high disagreement between maps here.

**Figure 2:**
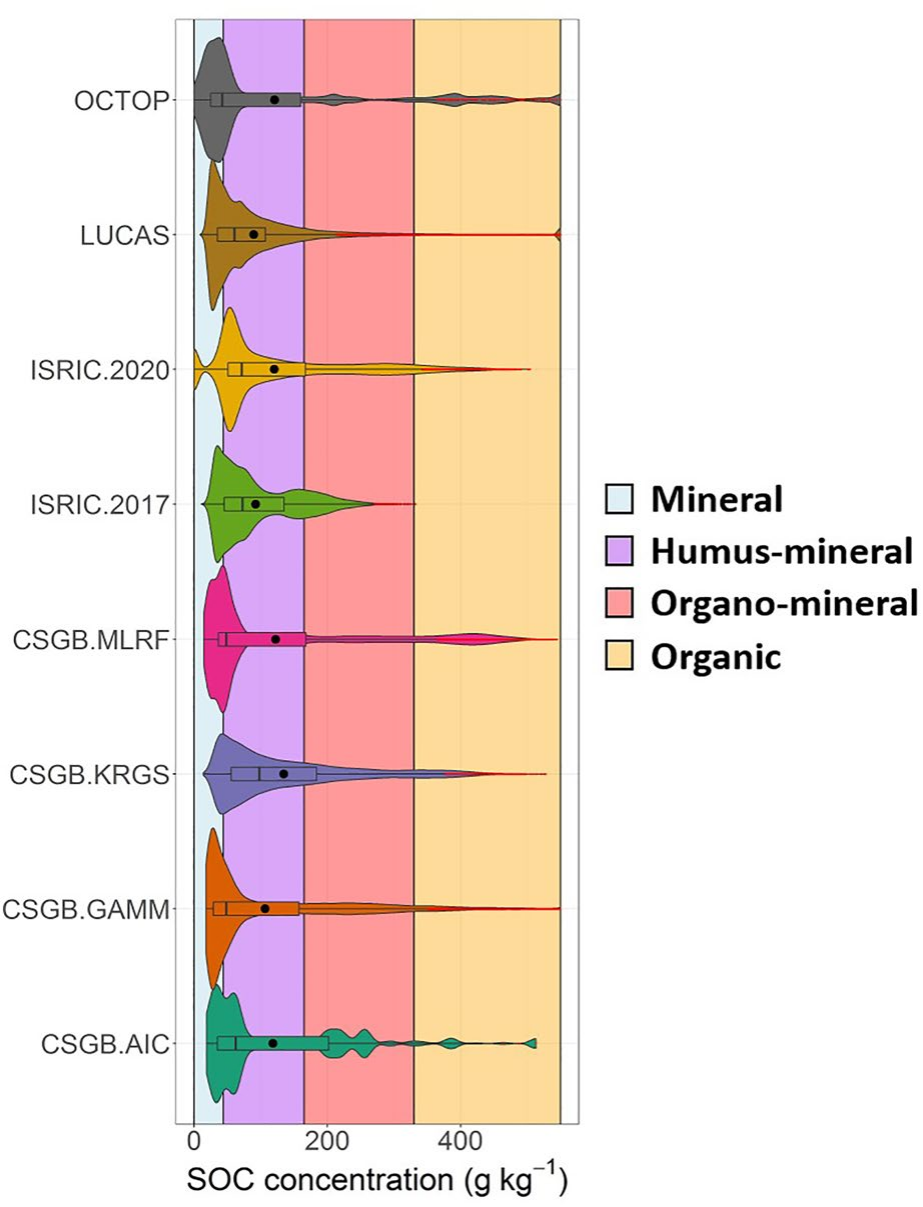
Distributions of modelled topsoil SOC concentrations for each map plotted as combined boxplots and violin plots (outliers in red), with coloured shading of the plot background denoting mineral (0–44 g kg^−1^), humus-mineral (44–165 g kg^−1^), organo-mineral (165–330 g kg^−1^) and organic (330–550 g kg^−1^) soils.

When plotted, the distributions of predicted topsoil SOC concentrations reveal broad similarities between the eight maps (Fig. 2). Each distribution is positively skewed with a heavy right tail. Most of the distributions are bimodal with a large peak < 100 g kg^−1^ representing mineral soils, and a secondary peak typically between 200 and 400 g kg^−1^ that appears to reflect the presence of high SOC content soils in the higher latitudes across all maps (Fig. 1a). The maps show similar predicted mean (~ 90–135 g kg^−1^) and median (~ 45–100 g kg^−1^) SOC concentrations to one another (Table S2). Pair-wise comparisons between the maps also reveal Pearson’s r coefficients ranging from 0.62 to 0.9, potentially indicating high levels of agreement overall (Figure S1).

However, it is possible to see marked differences between the maps. The LUCAS map for instance, exhibits a unimodal distribution of SOC concentration (Fig. 2). While most maps predict SOC concentrations up to 500–550 g kg^−1^, the maximum value predicted by ISRIC-2017 is only 332 g kg^−1^ (Table S2), with a secondary peak centred at ~ 150 g kg^−1^. Unlike the other maps, CSGB-KRGS predicts a lower proportion of mineral soils (0–44 g kg^−1^) and higher proportions of soils with intermediate SOC concentrations, including humus-mineral (44–165 g kg^−1^) and organo-mineral (165–330 g kg^−1^) soils.

An analysis of which map differs the most from the average of the eight maps at each 1 km grid location, reveals that the CSGB-KRGS, OCTOP and ISRIC-2020 maps tend to be the least conformative, particularly in Scotland, where the differences between the least conformative map and the eight-map average can be as high as 400% (Figure S2). These large discrepancies arise from one map predicting soils with mineral SOC concentrations where the other maps in our comparison suggest we would expect to find organic soils and vice versa.

### Evaluation of modelled topsoil organic carbon concentrations against survey data

**Figure 3:**
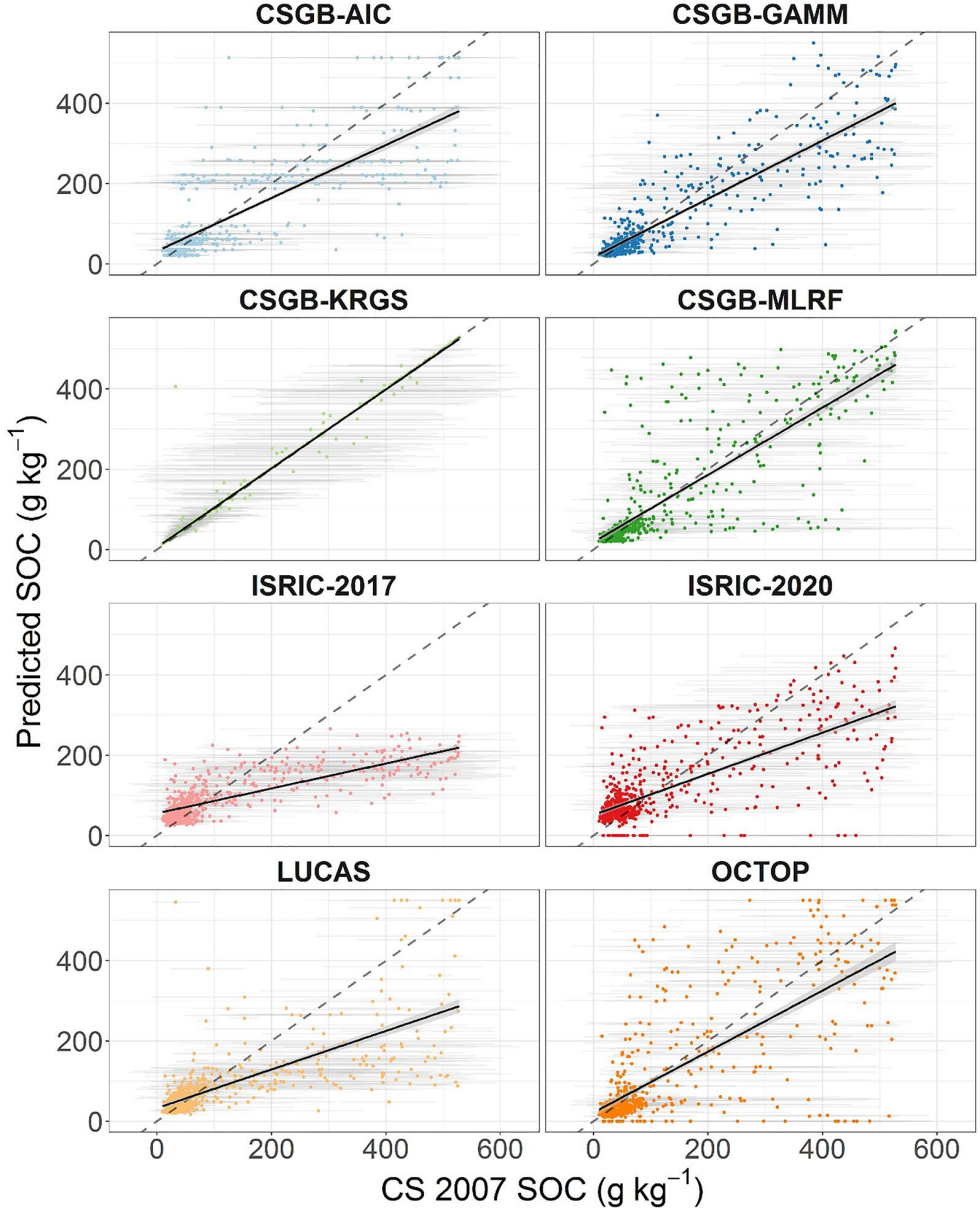
Predicted topsoil SOC concentrations versus measurements from the 2007 Countryside Survey (CS 2007) illustrated by scatter plots with 1:1 line (dashed) and best-fit linear model in black with grey shading representing ± 1 standard error of the mean. Grey horizontal lines around the points represent ± 1 standard error of the mean of the CS 2007 SOC concentrations.

A nationwide comparison shows that the four CSGB maps and OCTOP exhibit a near 1:1 relationship with CS 2007 survey observations (Fig. 3). The two ISRIC maps and the LUCAS map appear to predict higher concentrations than CS 2007 where these observations suggest low SOC contents. Conversely, lower concentrations are predicted where we would expect to find SOC-rich soils according to CS 2007 (Fig. 3). Except for the CSGB-KRGS map, there is a large degree of scatter, especially for observed intermediate SOC concentrations (30–300 g kg^−1^). This may suggest that the maps perform better at representing SOC concentrations at the extremes of the distribution (especially the low SOC mineral soils), but do not represent intermediate SOC concentration soils very well. On the other hand, in the case of CSGB-MLRF and OCTOP at least, where there is scatter for high observed SOC concentrations as well, the maps may have captured the expected range of values, albeit, not necessarily at the precise locations of the CS 2007 observations. However, except for the CSGB-KRGS map plot, all the best fitting linear relationships between CS 2007 observations and map predictions have a gradient well below 1 (Table S3). As a result, most maps under-predict SOC across a wide range of organo-mineral (> 165 g kg^−1^) and organic (> 330 g kg^−1^) SOC concentrations. Every map also over-predicts mineral (< 44 g kg^−1^) SOC concentrations, albeit to a much lesser extent. These patterns are not just confined to the comparison with CS 2007 data, with evaluation against LUCAS 2009 observations revealing similar over- and under-prediction tendencies among the maps (Figure S3; Table S3), which may suggest a common limiting factor in the construction of the maps themselves.

**Figure 4:**
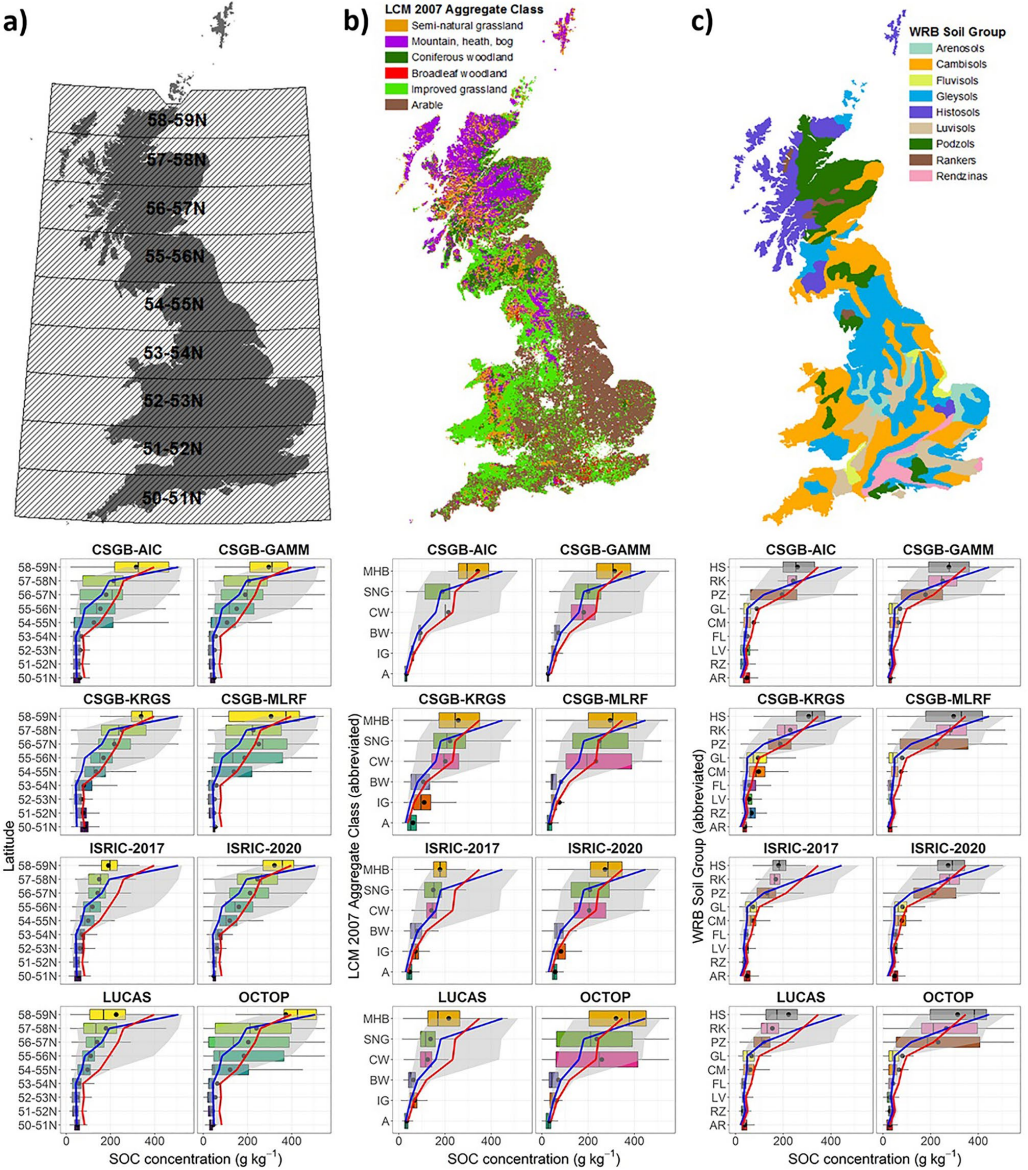
Topsoil SOC concentration distributions at the level of smaller-scale geographical units representing major environmental covariates, including at (**a**) each 1-degree latitude interval (excluding Orkney and Shetland due to the limited spatial coverage) to represent climate; (**b**) Land Cover Map (LCM) 2007^41^ Aggregate Class to incorporate effects of land management and organisms; and (**c**) the SoilGrids250m version 2 predicted major soil types, based on the World Reference Base (WRB) map from FAO/UNESCO^42^ to incorporate physical soil properties such as parent material. Boxplots are the topsoil SOC distribution modelled in all grid cells at each latitude interval for each of the eight maps (black circles are the means). The grey area is the IQR of the CS 2007 topsoil SOC distribution, and the solid blue and red lines denote the median and mean CS 2007 topsoil SOC values, respectively.

To investigate why differences or coincidences occur between map predictions, the map evaluations against CS 2007 data were stratified by different land types that represent major controls on SOC accumulation (see “Methods” section for further details). Comparing the distributions of CS 2007 values with the distributions of predicted SOC concentrations at these land type subsets, reveals clear differences in model performance between the maps (Fig. 4). For example, in areas containing high SOC concentrations according to CS 2007 (latitudes north of 56°N, semi-natural grassland and mountain, heath & bog land cover, and podzol, rankers and histosol soils) the LUCAS and ISRIC-2017 maps under-estimate mean, median and 75th percentile SOC concentrations considerably (Fig. 4). Conversely, the CSGB-MLRF and OCTOP maps in particular, capture similar concentration distributions to CS 2007 in these high SOC areas. One can also see clear improvements in the design of the SoilGrids250m model when comparing the ISRIC-2020 map with ISRIC-2017 (here, ISRIC-2020 distributions line up closer to the CS 2007 distributions than those of ISRIC-2017 do).

Similar patterns as described above are also visible in the Taylor Diagrams for each latitude band (Figure S4), land cover class (Figure S5) and major soil group (Figure S6). The LUCAS and ISRIC-2017 maps show the weakest fit to CS 2007 of all the maps across all Taylor Diagram statistics for most of the land types. The exception is the high standard deviation predicted by LUCAS in latitude 58-59 N, mountain, heath & bog land cover, and histosols, which we speculate may result from the high SOC concentrations predicted in the Hebrides and much lower concentrations predicted elsewhere at this latitude band, land cover type and soil group (see LUCAS map in Fig. 1b). Generally, the OCTOP and CSGB-MLRF maps match the standard deviation of observed SOC concentrations, while showing a higher RMSE and a lower Pearson’s R than the other maps. The CSGB-KRGS map appears to best reflect the observed SOC concentrations across all the Taylor Diagrams, though this is to be expected given the test data are identical to the training data for this map. CSGB-AIC, CSGB-GAMM and (to a lesser degree) ISRIC-2020, show intermediate levels of fit to CS 2007 across most of the Taylor Diagrams. Typically, these three maps do not perform as well as OCTOP or CSGB-MLRF at replicating the standard deviation of SOC observations, but often do exhibit slightly lower RMSE and higher Pearson’s r values.

## Discussion

Each map predicts the lowest SOC concentrations in the southeast, where low-lying mineral soils and agricultural land uses predominate, and predicts highest SOC concentrations towards the north and west of the country, where we might expect to find organic soils such as blanket peat in the cold, wet, high elevation areas (Fig. 1b). This results in maps predicting right-skewed distributions with a large proportion of mineral and humus-mineral SOC concentrations (< 100 g kg^−1^) and in a majority of cases, a smaller secondary peak (200–400 g kg^−1^) representing organo-mineral and organic SOC concentrations (Fig. 1a). Similar spatial correlations between SOC concentration and topographic and climatic conditions have been identified in published DSMs of France and North America^43,44^. As the coefficient of variation and signal to noise ratio maps illustrate, all the maps are in broad agreement with one another in the southern half of GB, where low SOC concentration mineral and humus-mineral soils predominate (Fig. 2). This might suggest that all eight maps perform excellently at capturing SOC conditions in agricultural areas where mineral and humus-mineral soils mainly occur. That said, one might expect scatter in these low SOC soils to be relatively small in part because low values are truncated by a natural asymptote of zero. Considerable disagreement exists in northern England, the Cambrian Mountains in Wales and in Scotland, which are dominated by blanket peat cover and other organic soils^45^. This is concerning as the higher SOC soils possess not just the greatest amount of SOC to lose, but are known to be the most susceptible to significant losses^12,46^. A lack of agreement among maps here raises challenges on how one might monitor the state and change of SOC storage in these SOC-rich soils.

The LUCAS and ISRIC-2017 maps fail to predict as high a proportion of organic SOC concentrations as we might expect, especially when compared with the CS 2007 observations (Fig. 3). The LUCAS map likely under-predicts SOC concentrations because of the bias towards mineral soils in the LUCAS 2009 dataset^15^. The creators of the LUCAS map themselves suggest that fitting a unimodal distribution to the training points from the LUCAS 2009 survey dataset, with a mean tilted towards low SOC concentrations associated with the mineral soils prevalent in this dataset led to systematic under-predictions in SOC concentrations across Europe^47^. The creators of the ISRIC-2017 map cross-validated their results against the WoSiS database, finding that the map under-estimated the overall mean of observed SOC concentrations^48^. This was likely driven by under-predicting the prevalence of organic soils and over-representing mineral soils. With the growth of the WoSiS database since the creation of the ISRIC-2017 map primarily, including the integration of LUCAS samples (see^17^), and secondarily, improvements in model calibration and cross-validation with the development of the ISRIC-2020 map^49,50^, we speculate that a smaller database of soil samples in WoSIS in 2016 compared to 2019 (also biased towards mineral soils), in conjunction with limitations in the fitted model drove the systematic under-prediction of SOC concentrations by ISRIC-2017.

Most maps did successfully model the presence of high SOC content organic soils as observed by the CS 2007 dataset (Fig. 3) and the LUCAS 2009 samples (Figure S3). The CSGB-GAMM map is similar to the LUCAS map in its construction, but used training points from CS 2007, which better represents organic soils than the LUCAS 2009 survey, and applied a Tweedie distribution for model fitting to account for the bimodal SOC distribution of GB^51^. The CSGB-AIC map was constructed via conventional upscaling (the process of combining soil property data from local observations with soil maps via class-matching and geo-matching^52^), using the best performing model (i.e. selecting the combination that minimised the value of Akaike’s Information Criterion when compared with CS 2007 data as the best-fit model) of broad habitat and parent material classes to predict SOC concentrations^53^. Consequently, the CSGB-GAMM and CSGB-AIC maps predicted the observed mean and median SOC concentrations of CS 2007 accurately when compared at the levels of individual latitude bands, soil types and land cover aggregate classes (Fig. 4). However, they were less effective at capturing the interquartile ranges or the standard deviations of observed SOC (Figure S4, S5 & S6), particularly where these were high for CS 2007, such as in the most northerly latitudes. Both of these maps were constructed using factor variables and CS 2007 survey square level averages of observed SOC, so these maps were unlikely to perform well when compared against individual CS 2007 sample points.

The CSGB-KRGS map predicted higher proportions of humus-mineral and organo-mineral soils at the expense of mineral and organic soils than any of the other maps (Fig. 1). CSGB-KRGS was generated using ordinary kriging to interpolate SOC concentrations between locations that were sampled as part of the CS 2007 survey^14^. While this resulted in an extremely good fit to the CS 2007 observations overall (Fig. 3), it should be noted that this is to be expected, given that the model data is the same as the averages of each CS 2007 survey square. Furthermore, kriging will simply smooth nearby observations, producing a larger proportion of predicted SOC of intermediate concentrations than we might expect to see on the ground^52^. Indeed, the large extent of land converted for agriculture, particularly in England, coupled with the large predicted SOC stock losses over the Holocene^46^ would suggest a high proportion of low SOC concentration soils. However, the smaller extent of blanket peat coverage in GB would produce a secondary peak of high SOC concentration organic soils (albeit, a smaller, but nevertheless pronounced peak given the relatively large proportion of blanket peat area in GB by global standards)^45^. The fact that CSGB-KRGS does not include environmental covariates when predicting SOC concentrations likely drives its failure to produce the bimodal distribution of SOC values described above.

When comparisons between maps and CS 2007 were broken down into smaller areas by latitude, land cover and soil type, the OCTOP and CSGB-MLRF maps appeared to capture the SOC concentration distributions (Fig. 4) and the variability of observed SOC (Figures S4, S5 & S6) of the CS 2007 dataset the best overall. A pedo-transfer rule (PTR), adapted from the original PTR 21^54^ was applied to a 1:1,000,000 scale harmonised spatial soil database of Europe to create the OCTOP map^55^. According to the creators of OCTOP, revisions to PTR 21 coupled with a highly detailed soil database allowed for more complete capture of high SOC content soils^55^. Due to differences in the sampling design of the soil surveys that make up the soil database the OCTOP mappers used (e.g. systematic sampling in England and Wales; clustered sampling in Italy), high SOC concentration areas defined in the database show large degrees of variation^55^. These high variations appear to be mirrored in the CS 2007 data for many of the latitudes, soil types and land cover aggregate classes we analysed. The CSGB-MLRF map’s reliance on a few core parameters to predict land cover, and subsequently SOM (which we converted to SOC g kg^−1^) likely helped to capture the high variability in observed SOC concentrations^56^.

Our study presents a relatively simple approach to comparing multiple published DSM products and analysing their differences. The approach outlined in this paper could be applied to other soil properties of interest, such as pH or bulk density, where several maps have also been published. Furthermore, the comparison could be expanded to include measurement of a much broader set of indicators. One study, comparing maps of soil pH in New York State, USA, quantified differences in whole-map statistics, visually-identifiable landscape features, level of detail, range and strength of spatial autocorrelation, landscape metrics (Shannon diversity and evenness, shape, aggregation, mean fractal dimension, co-occurrence vectors), and spatial patterns of property maps classified by histogram equalisation^57^. As additional maps are created in future, the focus will turn increasingly towards building ensemble maps. However, as we have shown, all of the maps in our study are similarly constrained in that they under-predict high SOC concentrations when evaluated against two different observational datasets (CS 2007 and LUCAS 2009). Thus, any attempt to create an ensemble map using datasets from our study would be similarly disadvantaged to the constituent primary maps that were used in its construction.

## Conclusion

Overall, our adoption of a multiple map comparison approach to analysing several national scale SOC concentration map products reveals broad alignment of predicted values in mineral soils, with progressively poor agreement in organo-mineral and organic soils. The maps we compared tended to show negative biases when evaluated against both CS 2007 and LUCAS 2009 observations, suggesting an over-prediction in the coverage of mineral soils at the expense of organic soils. A much larger range of higher SOC values are under-predicted by all maps, compared with the small range of carbon-poor soils that the maps over-predict compared to observational datasets. While we can partly explain this by pointing to biases in the underlying observational datasets or the methods used to generate distributions of predicted SOC concentrations, another factor may be the failure to adequately predict the locations and extents of peat soils. This under-estimation of the extents of organic soils is a major contributor to one of the outstanding gaps in our understanding of the land carbon-climate feedback identified by Crowther et al. (2016): “Uncertainty regarding current estimates of global soil C stocks”, particularly as the decline of SOC from global warming scales with the size of the standing SOC stocks^12^. This limitation is underscored by the fact that GB straddles a critical latitudinal gradient (~ 50-60°N) where SOC increases from ~ 1% in the south to ~ 100% in the north, where favourable conditions for peat formation prevail^27^. If this under-representation of organic soils was replicated elsewhere in this latitude range, it would have serious ramifications for quantifying SOC storage globally. Predicting the SOC concentrations of mineral soils accurately will also be important, particularly to assess soil structure and where actions such as organic matter addition need to be targeted. A recent analysis of soil survey data revealed that 38.2% of arable sites sampled in England and Wales contained at least 13 times as much clay as SOC, rating them as degraded^58^. Thus, urgent efforts are required to reduce these map uncertainties to increase confidence in SOC change detection at national and even up to global scales.

Going forward, further attention should be directed towards additional field surveys and ensuring these faithfully represent the full range of SOC concentrations in an area of interest (e.g. a country). Additional covariates that capture the convergence of physical factors that produce high SOC contents should be used in future DSMs, and statistical limitations need to be overcome to better distinguish between mineral and organic soils. As a stand-alone dataset, our map comparison results could be used to identify high risk areas for potential SOC loss by combining where the mean of the predictions is high (where carbon should be conserved) and uncertainty is also high (where monitoring should be targeted to close this gap in predictions).

## Methods

### Inter-comparison of eight topsoil organic carbon concentration maps

Eight maps of SOC concentration to a depth of 0–15 cm, 0–20 cm or 0–30 cm of topsoil, with coverage of GB were obtained and are summarised in Table 1. The maps cover a wide range of spatial scales, with four of these maps covering GB only and generated using the CS 2007 dataset; two covering most of Europe using EU-wide soil surveys and databases (LUCAS 2009 and the European Soils Database); and both versions of SoilGrids250m, generated from observations in the WoSIS database using machine learning methods, with global coverage published by the ISRIC World Soil Information site. SOC predictions were generated from a range of methods involving pedo-transfer rules, conventional upscaling, ordinary kriging, machine learning and statistical functions, and using a wide range of soil sample databases, remote sensing and other thematic layers as proxies for covariates of SOC. The two ISRIC SoilGrids250m maps also use tree-based machine learning algorithms (random forest and gradient tree boosting) to optimise SOC content predictions at depth by factoring in local relationships between soil variables and covariates^48^.

**Table 1:** Summaries of each of the eight topsoil SOC concentration maps.

Map; reference	Grid resolution (m); spatial coverage	Soil depth layers (cm)	SOC units	Prediction methods
ISRIC-2017^48^	250 (resampled by taking the area-weighted mean of all cells in a 1 km grid; see “Methods” section); global	0, 5 & 15 (converted to a single 0–15 layer using the trapezium rule)	dg kg^−1^ (divided by 10 to get to g kg^−1^)	Applied machine learning, including random forest & gradient boosting, to a harmonised global soil observation dataset (WoSIS), using 90% of observations for calibration; 10% for validation. Covariates used for model prediction include (but are not limited to): EVI, night & day-time land surface temperature, land cover, monthly precipitation, lithologic units, and multiple topographic variables
ISRIC-2020^49^	250 (resampled by taking the area-weighted mean of all cells in a 1 km grid; see “Methods” section); global	0–5 & 5–15 (converted to a single 0–15 layer by taking weighted means of the layers)	dg kg^−1^ (divided by 10 to get to g kg^−1^)	Same as above, but using: (1) A greater range of soil observations (updates to WoSIS soil database); (2) Improved model calibration & cross-validation; (3) Improved covariate selection & parameterisation; and (4) Prediction uncertainty quantified at the 90% prediction interval. Calibration on 90% of samples; validation on 10%
LUCAS^47^	500 (resampled by taking the area-weighted mean of all cells in a 1 km grid; see “Methods” section); Europe	0–20	g kg^−1^	Generalised additive model fitted to 85% of LUCAS 2009 survey points (15% used for validation), using slope, land cover, NPP, latitude & longitude as covariates for model prediction
OCTOP^55^	1000; Europe	0–30	% SOC (multiplied by 10 to convert to g kg^−1^)	Applied a pedo-transfer rule to all soil observations from the European Soil Database, with soil type, mean annual accumulated temperature, dominant surface textural class & land cover/use as covariates for model prediction. Validated using SOC data from Italy, England and Wales
CSGB-AIC^53^	1000; GB	0–15	g kg^−1^	Conventional upscaling/geo-matching to derive weighted-average SOC for different land units based on various combinations of land cover & parent material attributes. Inter-comparison of Akaike’s Information Criterion to judge model accuracies & to select the best map. Used all CS 2007 observations
CSGB-GAMM^51^	1000; GB	0–15	g kg^−1^	Applied a spatial GAMM modelling approach to all CS 2007 points. Covariates used included broad habitat class, soil group, CaCO_3_ rank, SO_4_, NH_4_ & NO_3_ deposition, 5-year means of seasonal temperature & precipitation. Validation by applying the model to LUCAS 2009 samples
CSGB-KRGS^14^	1000; GB	0–15	% LOI (multiplied by 5.5 as per^28^)	Interpolated a map of loss-on-ignition percentages from all CS 2007 sites (mean of 5 random points per square) using ordinary kriging. Sequential Gaussian simulation to estimate map uncertainty
CSGB-MLRF^56^	1000; GB	0–15	% LOI (multiplied by 5.5 as per^28^)	Applied a chain modelling approach of first using random forests to predict land cover from climate variables and then soil organic matter content from predicted land cover composition. Used CS 2007 in both modelling steps (80% of samples for model calibration; 20% for validation)

Prior to their comparison with one another, all the maps were harmonised to a common spatial extent, grid resolution and coordinate system. The two ISRIC maps (WGS84/EPSG:4326), LUCAS and OCTOP (both ETRS89 Lambert Azimuthal Equal Area/EPSG:2163) maps were converted from their native grid references to OSGB 1936/British National Grid (EPSG:27700). To ensure these maps would align exactly with the four Countryside Survey-based maps, the ISRIC-2017, ISRIC-2020, LUCAS and OCTOP maps were snapped to the CSGB-GAMM map as a reference. The choice of CSGB-GAMM as the reference grid is arbitrary and based purely on this map being the first one that we collected. This snapping process results in a simple translation that induces no resampling of raster grids that might distort the map data and ultimately our comparison results. The two ISRIC maps and the LUCAS map were resampled to 1 km spatial resolution using “Zonal Statistics” from the “Spatial Analyst Tools” in ArcGIS 10.6.1. We felt that using zonal statistics was more appropriate than the raster resampling methods for continuous data available in ArcGIS (“bilinear interpolation”, which takes the average of the surrounding 4 cells, and “cubic convolution”, which calculates the value of each pixel by fitting a smooth curve on the surrounding 16 pixels). A 1 km resolution vector grid represented the zones, and within each zone, the mean was taken of all four 500 m cell values of the LUCAS map and of all sixteen 250 m cell values of the ISRIC-2017 and ISRIC-2020 maps. The distinction between grid resolution and the scale of support should be emphasised here: For most maps the scale of support is a single soil observation core (e.g. for the LUCAS map, the scale of support is an individual 0–20 cm soil sample from the LUCAS 2009 survey). This means that at a particular grid cell, a map will show the expected SOC concentration for any topsoil observation sampled within this grid cell with a set of co-located covariates. CSGB-KRGS is the exception however, as all observations within a 1 km grid cell were averaged prior to spatial interpolation, therefore, the spatial support for this map is equivalent to the grid resolution of 1 km.

The two ISRIC maps contain SOC concentration data at different layers and require an additional processing step to obtain values for the 0–15 cm soil depth. For ISRIC-2017, SOC concentration data are recorded in 7 depth layers, including at 0, 5 and 15 cm. The mean SOC concentration at each grid cell was calculated for the 0–15 cm depth of soil by applying the trapezium rule to the 0, 5 and 15 cm layers as described in Hengl et al. (2017)^48^. For ISRIC-2020, SOC concentration data are recorded in depth intervals, including at 0–5 cm and 5–15 cm. Values for the 0–15 cm depth interval were calculated by taking a weighted average of the SOC concentrations of the 0–5 and 5–15 cm layers. All maps were compared using g kg^−1^ as the units for SOC concentration. The two ISRIC maps were converted from dg kg^−1^ by dividing by 10; the OCTOP map from % SOC by multiplying by 10; and the CSGB-KRGS and CSGB-MLRF maps from % LOI by multiplying by 5.5, based on the relationship in Eq. 1 in Reynolds et al. (2013) which suggests the theoretical limit of SOC in SOM is 55%^28^. Some maps (LUCAS, OCTOP and CSGB-GAMM) contained SOC concentrations exceeding the theoretical limit of ~ 55% of organic carbon in SOM. For these maps, all cells exceeding the theoretical maximum were set to a concentration of 550 g kg^−1^. The CSGB-AIC map contained a number of cells with negative SOC concentration values which were removed prior to comparison with other maps. All subsequent analyses and figure creations were undertaken using the R (ver. 4.0) programming language^59^.

As an initial comparison, the distributions of predicted SOC concentrations were plotted for each individual map. Next, focusing on just the grid cells that contain SOC concentration data from all 8 maps (n = 208,752), the mean, standard deviation, coefficient of variation (standard deviation/mean) and signal to noise ratio (mean/standard deviation) statistics were calculated to quantify the scale and spatial distribution of (dis)agreements between the maps. SOC concentrations of each map were subtracted from the mean of the eight-map collection and compared to identify which map stood out as the main driver of disagreements in predictions at each grid cell. Differences between the most deviant map and the mean of the eight maps at each grid cell were normalised by dividing the difference by the mean of the eight maps and multiplying by 100 to obtain percentages. The scale of the differences was also expressed in terms of the number of standard deviations of the mean that were exceeded (see maps in Figure S2b).

### Evaluation of modelled topsoil organic carbon concentrations against survey data

In order to assess how closely map predictions coincide with observations, each harmonised map was evaluated against the CS 2007 SOC concentration data (converted from loss-on-ignition (%) to g kg^−1^ by multiplying by 5.5) from the top 15 cm of soil. CS 2007 uses a stratified random sampling approach to cover forty-five landscape classes based on various topographic, geographic, geological and climatic features^14^. Sampling units of 1 km (n = 591) are sampled within each strata, within which five samples are taken at random (n = 2955) to account for short-range variation in soil properties including SOC^60^. This is particularly advantageous compared to other survey datasets because this probability sampling produces robust and unbiased estimates of statistical indicators such as the mean and variance^61^. This gives CS 2007 significant advantages over other available survey datasets. For example, the LUCAS 2009 dataset does not include samples taken in high elevation areas^15^, which leaves organic soils in much of Wales, northern England and Scotland poorly represented. The National Soils Inventory (NSI) of soils in England and Wales^62^ and in Scotland^63,64^ on the other hand, relies on systematic grid sampling at 5 km resolution, which does allow coverage of a far greater number of samples than CS 2007 (> 5500 points in England and Wales alone). However, these NSI datasets were not freely available, and according to Jones et al. (2005) in their evaluation of the OCTOP map, despite the large number of samples of NSI, the survey data tended to under-represent the presence of soils with low spatial coverage (including organic soils) in the country as a whole^55^. For the purpose of comparing the maps, we will assume that CS 2007, with its advantages over other soil surveys, represents the most accurate and complete picture of SOC concentrations available for GB.

For an initial evaluation against field observations, predicted SOC concentrations were extracted from all the maps to all of the CS 2007 points (n = 2614). As CS 2007 includes up to five randomly selected points per stratified survey location, the mean of each set of points in a 1 km square (including observations and extracted map predictions) was computed so map predictions could be compared with CS 2007 data. Map predictions were plotted against observations (Fig. 3). We also compared map predictions against observations from the LUCAS 2009 survey dataset to see whether similar patterns of systematic over- or under-prediction to our comparison of maps against CS 2007 occur (Figure S3). Lines of best fit for the relationships between map predictions and CS 2007 data, and between map predictions and LUCAS 2009 data were computed via linear regression. These lines of best fit were used to estimate the ranges of SOC concentrations that would be over-predicted and under-predicted by the maps compared to CS 2007 and LUCAS 2009 observations (Table S3).

Four of the maps were generated using CS 2007 data. While it is expected that these four maps would more closely align with CS 2007 observations than the other maps would, it is still useful to try to investigate differences between the CS 2007-based maps. It is also important that we emphasise that what we present here is not a validation study of several maps, but an investigation into what differences exist in map predictions and using a stratified random soil survey (which we argue is the most representative type of survey design for capturing the diversity of SOC contents across GB) to try to unpack the reasons why differences may have arisen.

Factors including climate, vegetation, land-use, micro-organisms, soil texture and parent material are likely significant controls on SOC dynamics at the spatial scale of GB^8^. These factors can be represented to a large extent by fractionating the land area of GB into 1-degree latitude bands (from 50 to 59°N), major soil types according to the World Reference Base (WRB) system (using the FAO/UNESCO vector map of WRB soil groups)^42^, and aggregate classes as defined in the 2007 UKCEH Land Cover raster map^41^. Due to the limited number of CS 2007 data points, we wanted to avoid fractionating the land surface too finely. SOC stocks have been shown to increase dramatically between the same latitude bands that GB occupies, ~ 50 and ~ 60°N^27^, providing further justification for stratifying by latitude bands. The 2007 Land Cover map was vectorised from its original GeoTiff raster format into polygons, with each polygon of the WRB and Land Cover maps representing a major soil type and land cover class, respectively. Graticules representing 1-degree interval latitude bands were downloaded from Natural Earth^65^, clipped to the extent of GB (excluding the Shetland and Orkney Islands) and vectorised. Map predictions at each 1 km grid were extracted to a grid of a points and intersected with thematic layers representing the three land types in vector format. The same intersection process was repeated with all the CS 2007 points (i.e. without taking an average of the five sample points per grid square as described in the previous paragraph). Distributions of all SOC observations from CS 2007 and predictions from all grid cells (n ~ 200,000; varies slightly by map) modelled by each of the maps were plotted for each stratum of each land type (e.g. each 1-degree latitude band for the stratification by latitudes) and compared (see Fig. 4). This comparison of map and CS 2007 distributions at finer subset spatial scales is useful for representing the range of SOC concentrations, but does not consider how well predicted SOC corresponds with CS 2007 at individual observation points. To address this, we produced Taylor Diagrams, using the ‘openair’ package in R^66^, comparing maps with CS 2007 SOC at each CS 2007 point for each latitude band, land cover class and major soil type. Taylor Diagrams incorporate three statistics on one 2D graph (the standard deviation, centred-RMSE and Pearson’s r correlation coefficient) by exploiting their relationship with one another, which can be represented via the Law of Cosines (see^67^ for more details on how Taylor Diagrams are constructed and interpreted). The results are plotted in Figures S4, S5 & S6. We chose not to compare map predictions with LUCAS 2009 survey points at the levels of individual latitude bands, land cover classes or soil types, due to the small sample size of this dataset for GB as a whole (n = 941), as well as its aforementioned limitations (e.g. no samples taken in elevations higher than 600 m above mean sea level).

## Supplementary Information

Supplementary Information.

## Data Availability

Some of our data, including code is available on request, though the Countryside Survey 2007 dataset contains sensitive information and cannot be readily shared.
