# Supplementary material for: Multiple soil map comparison highlights challenges for predicting topsoil organic carbon concentration at national scale
Source: Sci Rep. 2022 Jan 26;12:1379. doi: 10.1038/s41598-022-05476-5 (PMC8792051; doi:10.1038/s41598-022-05476-5)

Supplementary Information for

Multiple soil map comparison highlights challenges for predicting topsoil organic carbon concentration at national scale

Feeney, C. J.[1*], Cosby, B. J.[1], Robinson, D. A.[1], Thomas, A.[1], Emmett, B.[1], & Henrys, P.[2]

[1]UK Centre for Ecology and Hydrology, Environment Centre Wales, Bangor, UK.

[2]UK Centre for Ecology and Hydrology, Library Ave, Bailrigg, Lancaster, UK.

*Corresponding author: Christopher Feeney (chrfee@ceh.ac.uk)

This file includes:

Tables S1 to S3

Figures S1 to S6

Table S1: List of sources where maps were obtained

Map name	Source	Last accessed
CSGB-AIC	http://www.ukso.org/static-maps/countryside-survey-topsoil.html	2021-05-05
CSGB-GAMM	https://catalogue.ceh.ac.uk/documents/3aaa52d3-918a-4f95-b065-32f33e45d4f6	Shared by author: 2020-04-22; Available online since 2020-11-25
CSGB-KRGS	N/A, but details on its construction are available in Emmett et al. (2007)	Shared by author: 2020-06-09
CSGB-MLRF	N/A	Shared by author: 2020-10-16
ISRIC-2017	https://files.isric.org/soilgrids/former/2017-03-10/data/	2021-05-05
ISRIC-2020	https://soilgrids.org/	2021-05-05
LUCAS	https://esdac.jrc.ec.europa.eu/content/topsoil-soil-organic-carbon-lucas-eu25	2021-05-05
OCTOP	https://esdac.jrc.ec.europa.eu/content/octop-topsoil-organic-carbon-content-europe	2021-05-05

Figure S1: Pair-wise correlations between the topsoil SOC concentration maps. Plots running diagonally from the top-left to the bottom-right show the distribution of SOC concentration predicted by each map. Plots on the left show the relationship between pairs of maps. Pearson's r correlation coefficient values are displayed in the boxes on the right.

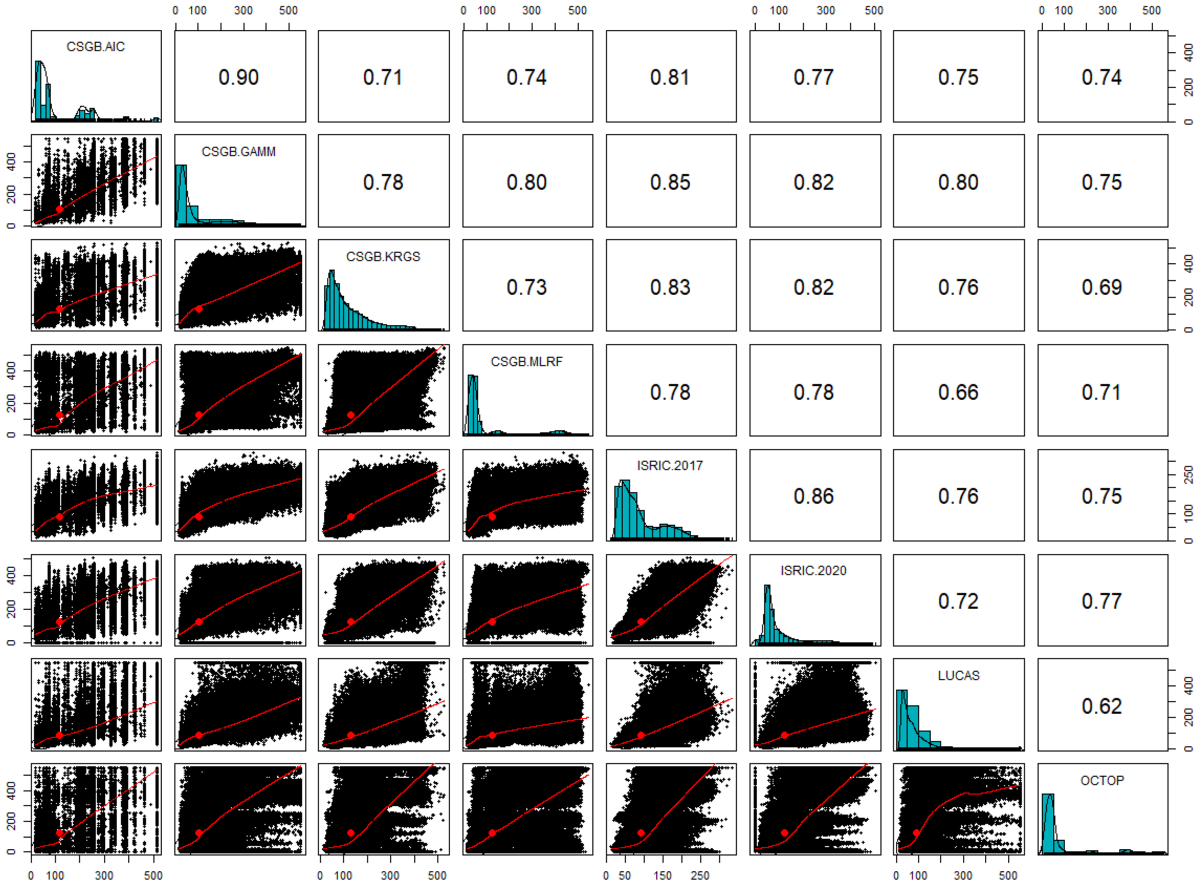

Table S2: Summary statistics of each map.

Map	CSGB-AIC	CSGB-GAMM	CSGB-KRGS	CSGB-MLRF	ISRIC-2017	ISRIC-2020	LUCAS	OCTOP
Mean	118.21	106.52	134.80	122.65	92.57	120.33	89.66	120.81
Minimum	18.94	17.89	13.97	14.60	10.83	0.00	9.02	0.00
Maximum	513.03	550.00	527.58	544.74	332.00	503.90	550.00	550.00
Standard Deviation	118.66	114.41	102.23	137.67	58.74	105.86	90.41	154.11
Skewness	1.52	1.60	1.21	1.37	0.93	1.23	2.93	1.51
Kurtosis	1.69	1.78	0.61	0.40	-0.15	0.49	10.45	0.86
Q5	21.43	22.00	33.73	21.64	29.83	0.00	23.60	10.08
Q10	25.31	23.56	38.43	22.98	33.17	35.47	25.90	14.32
Q15	32.42	25.30	43.61	24.47	36.67	43.43	28.58	18.19
Q20	35.19	27.12	49.51	27.60	40.83	47.60	31.74	22.58
Q25	35.19	29.14	55.98	36.87	45.33	50.77	35.13	24.96
Q30	35.19	31.78	62.71	39.53	50.17	53.87	39.08	29.09
Q35	49.04	35.68	69.93	41.64	55.17	57.30	43.24	34.45
Q40	52.91	39.83	78.02	43.54	60.50	61.07	48.07	37.57
Q45	60.02	43.97	87.20	45.60	66.50	65.53	53.81	40.18
Q50	62.80	48.59	98.17	48.29	72.67	71.63	60.63	42.42
Q55	62.80	54.82	110.85	52.61	79.00	81.30	67.69	46.82
Q60	62.80	62.57	125.84	59.37	86.33	94.97	74.38	52.40
Q65	81.51	78.55	143.04	68.30	96.83	112.40	82.97	59.09
Q70	186.66	110.17	162.21	122.49	113.50	135.13	93.56	91.03
Q75	201.97	157.36	183.86	167.97	135.00	167.33	106.82	160.24
Q80	221.43	200.95	211.20	245.08	152.00	212.87	122.96	220.38
Q85	254.78	241.74	249.09	319.10	167.50	259.60	144.86	356.49
Q90	257.56	286.55	303.23	387.15	185.00	299.83	178.90	397.20
Q95	381.42	357.86	362.59	429.77	207.67	347.10	259.16	464.88
Q99	513.03	483.29	421.95	476.83	247.50	418.29	550.00	550.00

Figure S2: Maps which deviate the most from the average of the ensemble. a) Percentages of grid cells where each of the eight maps is the most deviant from the ensemble mean displayed in Figure 2 in the main text and sub-divided into over-estimates and under-estimates relative to the mean of the eight-map collection. b) Spatial distributions of where each map happens to disagree the most with the mean of the 8 maps, with the scale of the disagreements expressed, both as a percentage of the mean SOC concentration of the 8 maps, and as the number of standard deviations of the mean this level of disagreement exceeds (1 [red] or 2 [blue]) in a given 1 km grid cell. Note here that statistics were calculated after each of the 8 maps were modified to harmonise them to a common spatial extent, resolution and units of SOC concentration. Statistics were only calculated where data exists from all 8 maps.

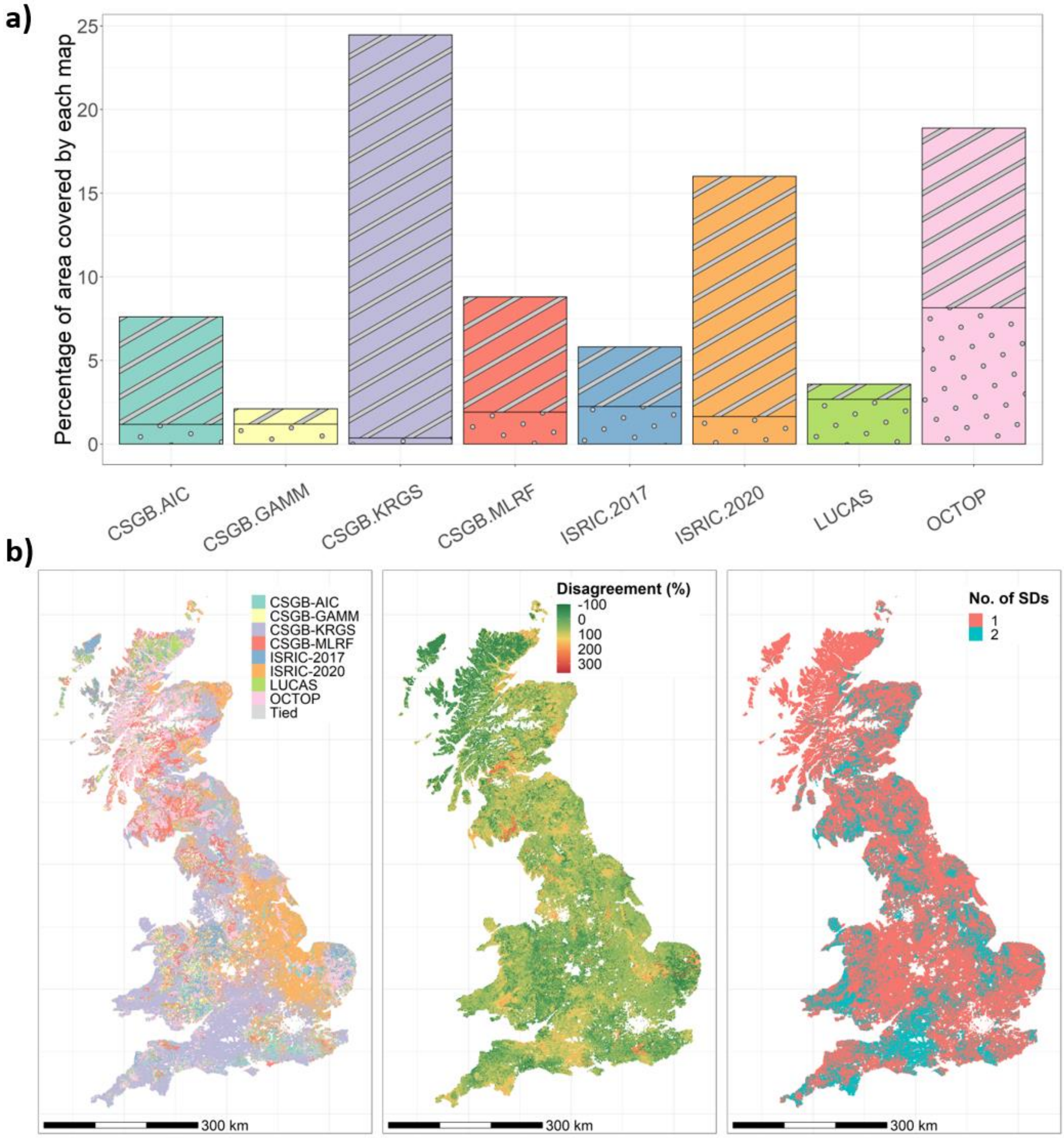

Figure S3: Predicted topsoil SOC concentrations versus measurements from the LUCAS 2009 field survey dataset illustrated by scatter plots with 1:1 line (dashed) and best-fit linear model in black with grey shading representing ±1 standard error of the mean.

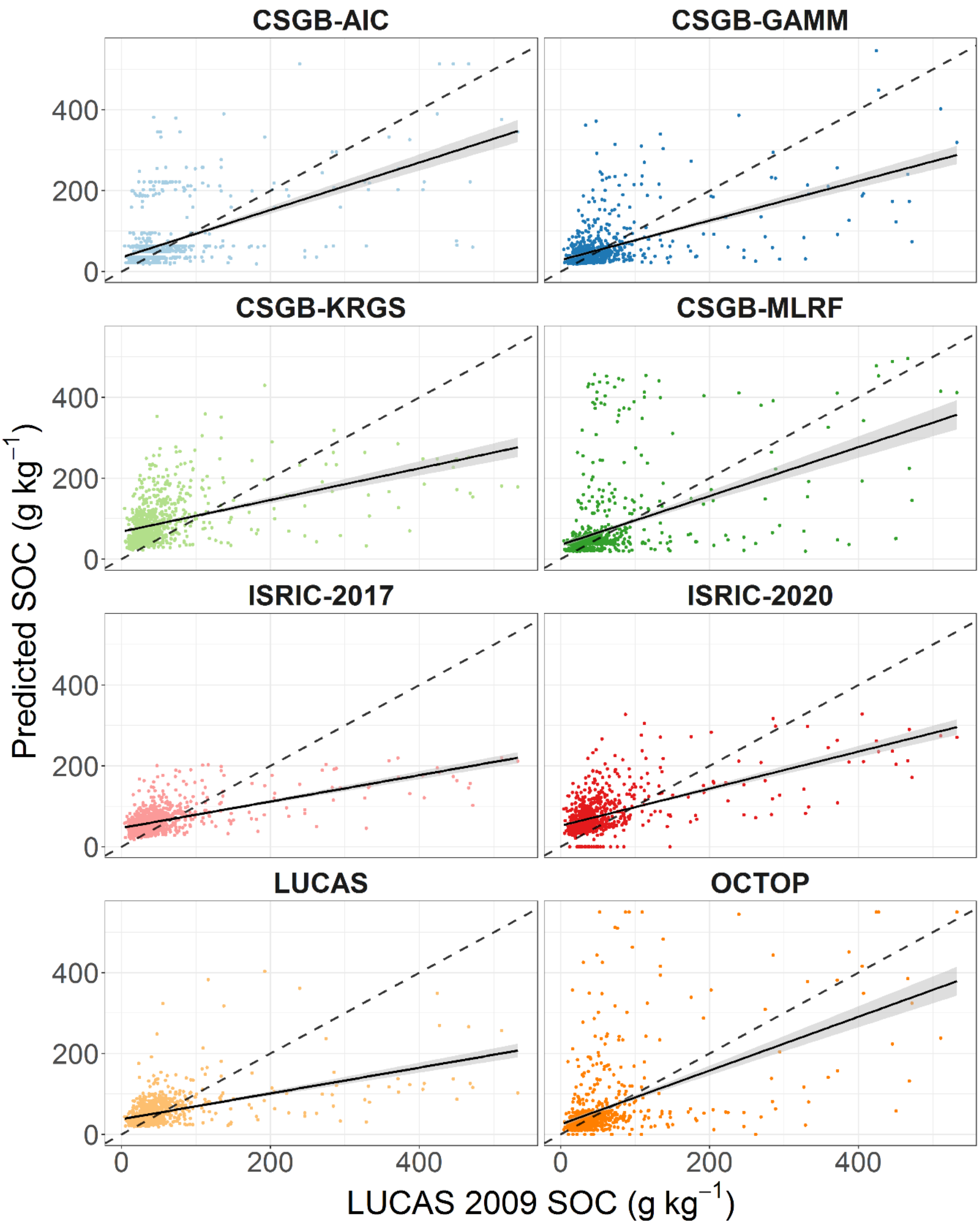

Table S3: Statistics calculated for the lines of best-fit for map predictions vs CS 2007 and map predictions vs LUCAS 2009. Included here are the ranges of SOC concentrations (rounded to the nearest integer) that are under- and over-predicted.

SOC Map	Intercept	Gradient	Adjusted R-squared (goodness of fit)	Over-estimated SOC Range (g/kg)	Under-estimated SOC Range (g/kg)
	CS 2007				
CSGB-AIC	32.20	0.66	0.66	<94	>94
CSGB-GAMM	17.48	0.72	0.76	<63	>63
CSGB-KRGS	6.02	0.98	0.98	<339	>339
CSGB-MLRF	18.91	0.84	0.68	<116	>116
ISRIC-2017	55.43	0.31	0.64	<80	>80
ISRIC-2020	51.39	0.51	0.56	<105	>105
LUCAS	32.98	0.48	0.50	<63	>63
OCTOP	21.77	0.76	0.54	<90	>90
	LUCAS 2009				
CSGB-AIC	35.02	0.59	0.32	<84	>84
CSGB-GAMM	28.64	0.49	0.33	<55	>55
CSGB-KRGS	68.09	0.39	0.21	<111	>111
CSGB-MLRF	35.57	0.60	0.22	<89	>89
ISRIC-2017	46.84	0.33	0.37	<69	>69
ISRIC-2020	52.37	0.46	0.37	<96	>96
LUCAS	37.90	0.32	0.27	<55	>55
OCTOP	25.26	0.66	0.26	<75	>75

Figure S4: Taylor diagrams per latitude band of map-predicted SOC distributions versus observed SOC distributions at each individual CS 2007 point.

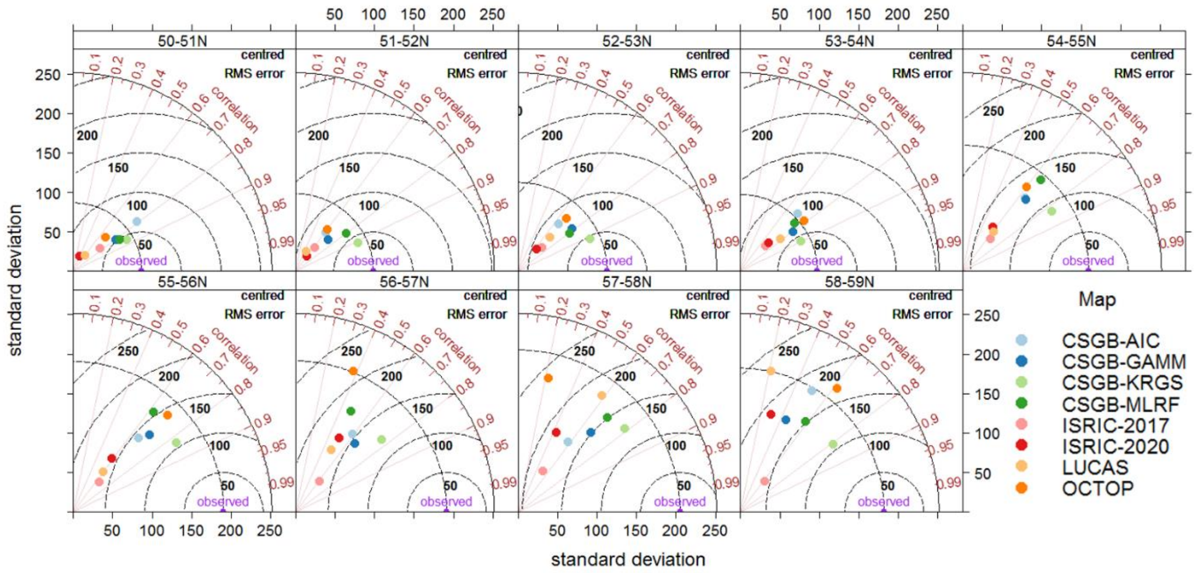

Figure S5: Taylor diagrams per land cover aggregate class of map-predicted SOC distributions versus observed SOC distributions at each individual CS 2007 point.

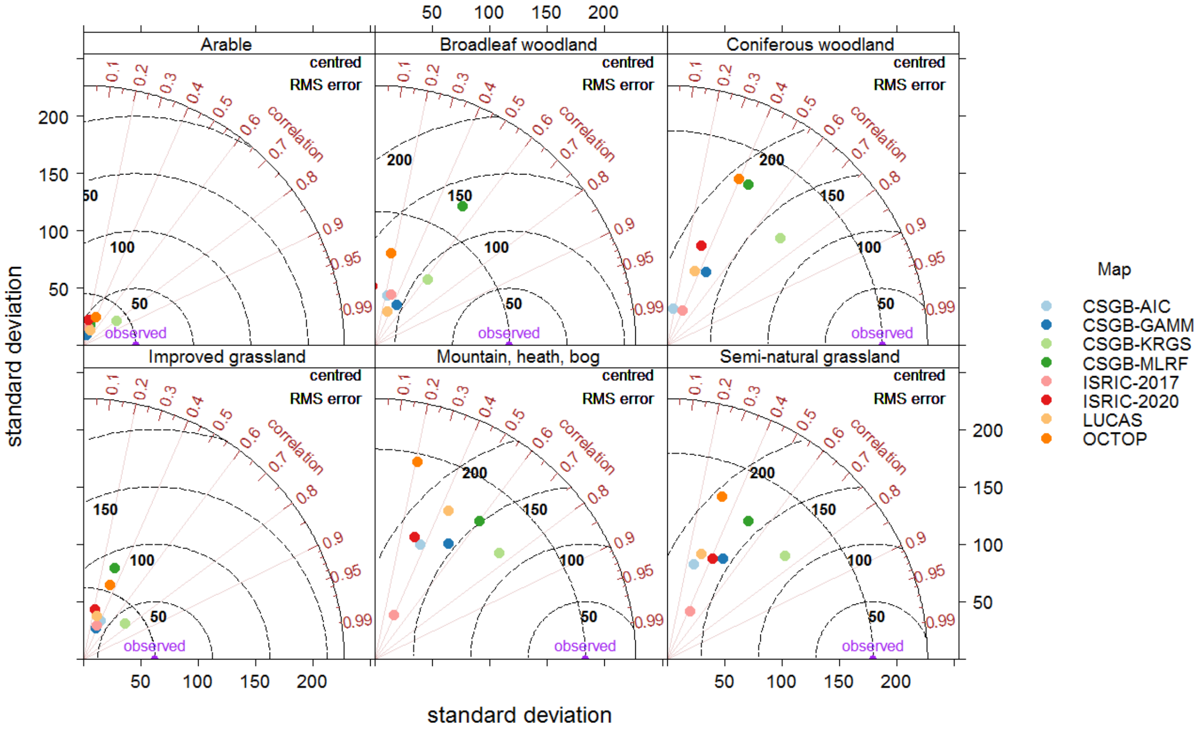

Figure S6: Taylor diagrams per World Reference Base major soil type of map-predicted SOC distributions versus observed SOC distributions at each individual CS 2007 point.

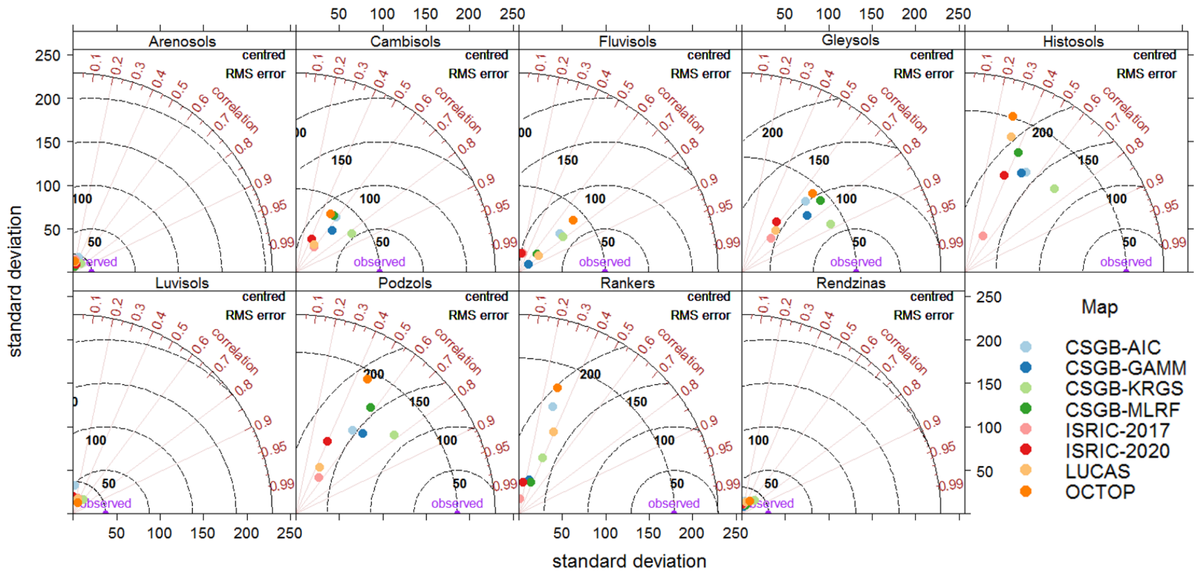

Supplement: Supplementary file 1 — Supplementary Information. [file 41598_2022_5476_MOESM1_ESM.pdf]
